# Production of (R)-3-hydroxybutyric acid by *Arxula adeninivorans*

**DOI:** 10.1186/s13568-016-0303-z

**Published:** 2017-01-03

**Authors:** Mateusz Biernacki, Jan Riechen, Urs Hähnel, Thomas Roick, Kim Baronian, Rüdiger Bode, Gotthard Kunze

**Affiliations:** 1 Leibniz Institute of Plant Genetics and Crop Plant Research (IPK), Correnstr. 3, 06466 Gatersleben, Saxony-Anhalt Germany; 2 Jäckering Mühlen- und Nährmittelwerke GmbH, Vorsterhauser Weg 46, 59007 Hamm, Germany; 3 School of Biological Sciences, University of Canterbury, Private Bag 4800, Christchurch, New Zealand; 4 Institute of Microbiology, University of Greifswald, Jahnstr. 15, 17487 Greifswald, Germany

**Keywords:** (R)-3-HB, *Arxula adeninivorans*, β-Ketothiolase, Acetoacetyl-CoA reductase

## Abstract

(R)-3-hydroxybutyric acid can be used in industrial and health applications. The synthesis pathway comprises two enzymes, β-ketothiolase and acetoacetyl-CoA reductase which convert cytoplasmic acetyl-CoA to (R)-3-hydroxybutyric acid [(R)-3-HB] which is released into the culture medium. In the present study we used the non-conventional yeast, *Arxula adeninivorans*, for the synthesis enantiopure (R)-3-HB. To establish optimal production, we investigated three different endogenous yeast thiolases (Akat1p, Akat2p, Akat4p) and three bacterial thiolases (atoBp, thlp, phaAp) in combination with an enantiospecific reductase (phaBp) from *Cupriavidus necator* H16 and endogenous yeast reductases (Atpk2p, Afox2p). We found that *Arxula* is able to release (R)-3-HB used an existing secretion system negating the need to engineer membrane transport. Overexpression of *thl* and *phaB* genes in organisms cultured in a shaking flask resulted in 4.84 g L^−1^ (R)-3-HB, at a rate of 0.023 g L^−1^ h^−1^ over 214 h. Fed-batch culturing with glucose as a carbon source did not improve the yield, but a similar level was reached with a shorter incubation period [3.78 g L^−1^ of (R)-3-HB at 89 h] and the rate of production was doubled to 0.043 g L^−1^ h^−1^ which is higher than any levels in yeast reported to date. The secreted (R)-3-HB was 99.9% pure. This is the first evidence of enantiopure (R)-3-HB synthesis using yeast as a production host and glucose as a carbon source.

## Introduction

Production of chiral compounds is an area of interest not only for pharmaceuticals but also in the fine chemicals industry. Synthesis of these compounds can be carried out either chemically or biologically. The fact that stereoisomers can differ in physical, chemical and biological properties is important and because of this, racemic mixtures have to be separated before use in industrial products and drug synthesis. However biological synthesis can have an important advantage over chemical synthesis in that only one of the stereoisomers may be produced. Additionally, chemical synthesis requires expensive catalysts, toxic solvents and harsh conditions, whereas biological synthesis of stereoisomers occurs in an aqueous environment under mild conditions.

A number of microbiological syntheses have been developed to produce chiral compounds (Goldberg et al. 2007; Johanson et al. 2005). One of these is (R)-3-hydroxybutyric acid [(R)-3-HB], which is the monomer of the polymer polyhydroxybutyrate (PHB). (R)-3-HB has applications as a nutrition source and as a precursor for vitamins, antibiotics and pheromones (Sutter and Seebach 1983; Chen and Wu 2005; Tasaki et al. 1999). Recently (R)-3-HB has been investigated as a potential protective agent against neuronal death (Julio-Ampilas et al. 2015).

Microbiologically (R)-3-HB can be synthesized in two pathways. One of these includes synthesis of PHB followed by enzymatic depolymerisation to obtain (R)-3-HB. However, this method requires a two-step fermentation process, which is more expensive to operate (Shiraki et al. 2006). The second approach includes the direct synthesis of (R)-3-HB through condensation of two acetyl-CoA molecules to acetoacetyl-CoA by β-ketothiolase followed by reduction of the latter to 3-hydroxybutyrate-CoA. Acetoacetyl-CoA reductase, the enzyme which catalyzes second conversion, is responsible for the stereoselectivity. The reductase, phaBp from *Cupriavidus necator*, produces the (R) form while the reductase, hbdp from *Clostridium acetobutylicum*, synthesizes the (S) form (Tseng et al. 2009). The final steps involve removing the CoA group and secretion of the (R)-3-HB into the culture medium.

Processes described in the literature usually employ overexpression of two first enzymes and various systems for improving excretion step. Matsumoto et al. (2013) demonstrated production of (R)-3-HB from glucose at 5.2 g L^−1^ by overexpression of *phaA* and *phaB* genes from *C. necator* and *pct*, which encodes propionyl-CoA transferase from *Clostridium propionicum*, using *Escherichia coli* as a host organism. Employing another secretion system with *tesB* gene (encoding *E. coli* thioesterase II), Liu et al. (2007) produced up to 12 g L^−1^ of (R)-3-HB. To date the maximal level of secreted (R)-3-HB achieved in a single fermentation process is 40.3 g L^−1^ using the bacteria *Halomonas* sp. KM-1 (Kawata et al. 2014). Recently, *Saccharomyces cerevisiae* has been reported as the first yeast to produce the second enantiomer form—(S)-3-HB. Yun et al. (2015) achieved 12 g L^−1^ of (S)-3-HB after a 220 h fed-batch fermentation using ethanol as the carbon source. However there are no reports describing the production of (R)-3-HB using yeast which use a simple carbon source such as glucose.

The non-conventional yeast *Arxula adeninivorans*, previously described as a valuable platform for production of recombinant proteins (Rauter et al. 2014; Chamas et al. 2015; Jankowska et al. 2013) and as a producer of the copolymer PHB-V (Terentiev et al. 2004), was proposed for the synthesis of (R)-3-HB. The advantages of using *A. adeninivorans* as a host are its wide substrate spectrum, its robustness and non-pathogenicity (Lindenkamp et al. 2012). Another advantage for using *A. adeninivorans* is that the wild type does not secrete (R)-3-HB but as demonstrated in this article, it has an endogenous secretion system that will export (R)-3-HB. There are cytosolic and mitochondrial thiolases in *A. adeninivorans* which could be used for (R)-3-HB production. Further there are two potentially useful reductases: Afox2p which is a peroxisomal multifunctional enzyme involved in fatty acid oxidation and known to catalyze the synthesis of the (R) form in *S. cerevisiae* (Hiltunen et al. 1992), and Atpk2p which is predicted to be mitochondrial 3-hydroxybutyryl dehydrogenase.

Here we describe the production of enantiopure (R)-3-HB by overexpression of genes encoding β-ketothiolase and acetoacetyl-CoA reductase in the yeast *A. adeninivorans.*

## Materials and methods

### Strains and cultivation condition

For cloning experiments and plasmid isolation, *E. coli* XL1 Blue [*recA1 endA1 gyrA96 thi*-*1 hsdR17 supE44 relA1 lac* [F′ *proAB lacI* ^*q*^ *Z∆M15* Tn*10* (Tet^r^)]], obtained from Invitrogen, was used. Growth media was Luria–Bertani (LB—Sigma, USA) supplemented with 100 mg L^−1^ ampicillin, 50 mg L^−1^ chloramphenicol or 50 mg L^−1^ kanamycin.

The wild-type strain *A. adeninivorans* LS3, originally isolated from wood hydrolysate in Siberia and deposited as *A. adeninivorans* SBUG 724 into the strain collection of the Department of Biology of the University of Greifswald (Kunze and Kunze 1994) was used as a control strain. The auxotrophic mutant, *A. adeninivorans* G1216 [*aleu2 ALEU2::aade2*] (Alvaro-Benito et al. 2012), was used as recipient strain. All strains were cultivated at 30 °C, 180 rpm in 200 mL of broth in a 1-L flask. The medium was either selective yeast minimal medium supplemented with 50 g L^−1^ glucose and 43 mM NaNO_3_ (YMM-glc-NO_3_) (Tanaka et al. 1967; Rose et al. 1990) or non-selective yeast complex medium with the addition of 30 g L^−1^ glucose (YPD).

To investigate (R)-3-HB utilization, appropriate strains were first cultivated in YMM-glc-NO_3_ (2% glucose) and, after 36 h incubation at 30 °C cultures were centrifuged and cell pellets were washed with YMM-NO_3_ (without glucose). Cells were then resuspended in YMM-NO_3_ media with 0, 0.1 and 0.2% (R)-3-HB as a carbon source with starting OD_600_ = 20. The cultures were incubated at 30 °C for 48 h and (R)-3-HB and dry cell weight were measured at intervals over the incubation period.

Fed-batch cultures were performed in a 5-L fermenter (Sartorius, Germany) with conditions set to optimize growth and (R)-3-HB production. The temperature was maintained at 30 °C and a pH of 6.0 was controlled by the addition of 2.5 M NaOH or 1 M H_2_SO_4_. The level of oxygen was established at pO_2_ = 1% (glucose feeding) or pO_2_ = 40% (ethanol feeding) and maintained by the stirring rate. The culture was started in YPD followed by the addition of glucose and nitrogen to prevent the depletion of glucose and to maintain the metabolism of the organism. Foaming was reduced by the controlled addition of a silicone-based anti-foam agent (Strunktol SB 304, Schill + Seilacher GmbH, Germany).

### Plasmid construction

The open reading frames (ORFs) of the bacterial genes were synthesized by GeneArt (Life Technologies) using optimized codon usage. ORFs originating from *A. adeninivorans* were obtained by PCR using genomic DNA of the wild type LS3 strain as a template and suitable primers (Table 1). One of the enzymes, Akat4p, was predicted (MITOPROT http://ihg.gsf.de/ihg/mitoprot.html) to be a mitochondrial protein. To obtain a cytoplasmic protein, ORFs without predicted leader sequence (*AKAT*_*N10*_ and *AKAT*_*N17*_ version of basic *AKAT4* gene) were amplified as described above using additional primers. All complete ORFs were inserted into pBS-TEF1-PHO5-SA vector with pair of restriction sites to obtain expression modules containing the *A. adeninivorans* derived *TEF1* strong constitutive promoter and the *S. cerevisiae PHO5* terminator (Böer et al. 2009). A set of primers (Table 1) and the above plasmids were used in PCR to amplify the DNA fragments with an ORF, promoter, terminator and additional restriction sites. A two-step cloning procedure was used for the construction of the final expression plasmid based on Xplor2.4 system (Alvaro-Benito et al. 2012). The reductase gene (*phaB* [LT608132]) was used to make the TEF1-phaB-PHO5 fragment flanked by 5′-*Bsi*WI/*Spe*I and 3′-*Mlu*I/*Sac*II sites and was introduced into the basic vector to create Xplor2.4-TEF1-phaB-PHO5. Subsequently the expression module containing the *TEF1* promoter, *PHO5* terminator, different versions of *β*-*ketothiolase* gene (*atoB* [LT608129], *thl* [LT608130], *phaA* [LT609131], *AKAT1* [LT608124], *AKAT2* [LT609125], *AKAT4* [LT608126], *AKAT4*_*N10*_ [LT608127], *AKAT4*_*N17*_ [LT609128]) and 5′-*Spe*I and 3′-*Bsi*WI restriction sites was cloned into the vector and final expression plasmids Xplor2.4-TEF1-thiolase-PHO5-TEF1-phaB-PHO5 were generated. Additionally, for the genes of endogenous reductases (*ATPK2* [LT632551], *AFOX2* [LT632550]) plasmids Xplor2.4-TEF1-thl-PHO5-TEF1-ATPK2-PHO5 and Xplor2.4-TEF1-thl-PHO5-TEF1-AFOX2-PHO5 were prepared in the same procedure.

**Table 1:** Oligonucleotide primers used for the construction of expression plasmids

Designation	Oligonucleotide sequence	Source
ORFs primers		Eurofins-Genomics
Akat1_start_MunI	TAGA*CAATTG*ATGGACAGACTCAACAACGTAGC	
Akat1_stop_BamHI	GCAA*GGATCC*TTACTCCCGGATAAACAGAGC	
Akat2_start_BamHI	TAGA*GGATCC*ATGGAAAGAGCATCTAATCTTGCC	
Akat2_stop_NotI	GCAA*GCGGCCGC*CTACTCCCTAATGAAGAGCG	
Akat4_start_EcoRI	TAGA*GAATTC*ATGTCTGTTTATATTCTTAGTGCTAAG	
Akat4_N10__start_EcoRI	TAGA*GAATTC*ATGACACCCATTGGTTCGTTTTTGGG	
Akat4 _N17__start_EcoRI	TAGA*GAATTC*ATGGGATCTCTGTCTTCTCAGAC	
Akat4_stop_BamHI	GCAA*GGATCC*TCAAACTCTCTCAATTACAAGAGC	
Atpk2_start_BamHI	GCAA*GGATCC*ATGCTACGACGGGGAATTC	
Atpk2_stop_NotI	TA*GCGGCCGC*TCACAACTTTGCCCCAGC	
Afox2_start_EcoRI	TAGA*GAATTC*ATGTCGGTGCCAACGGC	
Afox2_stop_NotI	TA*GCGGCCGC*TTAAAGCTTGGCTCCTCCG	
Two-step cloning		
TEF1_SpeI	TATA*ACTAGT*TAGTAGCGCTAATCTATAATCAG	
PHO5_BsiWI	CGGA*CGTACG*AGCTTGCATGCCTGCAGA	
TEF1_SpeI_BsiWI	TAT*ACTAGT*ACTT*CGTACG*CTCGACTTCAATCTATAATCAGTC	
PHO5_SacII_MluI	GGAT*CCGCGG*CCGA*ACGCGT*AGCTTGCATGCCTGCAGATTTTAATC	

Restriction sites were designated in italic

**Fig. 1:**
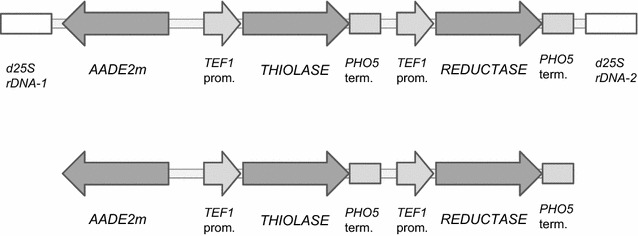
Physical map of DNA transformed to auxotrophic *A. adeninivorans* G1216 strain for (R)-3-HB production. Fragments were obtained after linearization with *Asc*I (fragment included d25S rDNA sequences) or *Sbf*I (without d25S rDNA). *THIOLASE* is referred to *atoB/thl/phaA/AKAT1/AKAT2/AKAT4/AKAT4*_*N10*_ */AKAT4*_*N17*_ genes encoding different versions of β-ketothiolases and *REDUCTASE* gene to *phaB/ATPK2/AFOX2* encoding acetoacetyl-CoA reductases. Both genes were fused with *TEF1* promoter and *PHO5* terminator to obtain two expression modules

All variants of the final plasmids were linearized with *Asc*I or *Sbf*I restriction enzymes and separately transformed into the auxotrophic strain, *A. adeninivorans* G1216 (Fig. 1).

### DNA manipulation and transformation

*Escherichia coli* and *A. adeninivorans* cells were transformed as described previously (Böer et al. 2009). Stabilization of yeast transformants was performed with passaging on selective and non-selective media according to Klabunde et al. (2003). Isolation of plasmid DNA and DNA restrictions were carried out as described by Wartmann et al. (2003).

### Enzyme activity

Both enzymatic activity assays were performed using permeabilized cells. Transformants were cultivated in 10 mL YMM-glc-NO_3_ medium for 48 h, centrifuged and the cell pellet was resuspended in 100 mM Tris–HCl pH 8.1 (or 50 mM phosphate buffer pH 7.4 + 1 mM DTT for the reductase assay) with 0.1% Triton X-100 and frozen at −20 °C. After thawing on ice, the cells were washed twice with buffer without Triton X-100 and the OD_600_ was adjusted to 1 before using in the assay.

#### β-Ketothiolase

Enzymatic activity was determined spectrophotometrically in the thiolysis direction using a modified version of the method described by Lindenkamp et al. (2012). The reaction mixture contained 100 mM Tris–HCl (pH 8.1), 25 mM MgCl_2_, 100 μM CoA and 40 μM acetoacetyl-CoA; reaction was initiated with cells and carried out for 15 min at 30 °C. After incubation, the reaction mixture was centrifuged at 3000×*g* for 1 min and the supernatant was analyzed spectrophotometrically (Infinite M200, TECAN). The decrease of the peak height was due to the disappearance of magnesium chelated acetoacetyl-CoA and was observed at 303 nm. The concentration was calculated using the extinction coefficient of acetoacetyl-CoA (16.5 mM^−1^ cm^−1^). Reactions without cells were incubated on ice and used as a blank.

One unit of β-ketothiolase activity was defined as the proportion of 1 nmol acetoacetyl-CoA that was cleaved to acetyl-CoA in 1 min by 1 mg of dry permeabilized cells.

#### Acetoacetyl-CoA reductase

A reductase activity assay was performed according to Kim et al. (2014) with slight modifications. The reaction mixture contained 50 mM phosphate buffer (pH 7.4), 1 mM DTT, 100 μM acetoacetyl-CoA and 200 μM NADPH. After addition of permeabilized cells the reaction mixture was incubated for 30 min at 30 °C, processed as above and oxidation of NADPH was monitored at 340 nm. The extinction coefficient of NADPH was 6.22 mM^−1^ cm^−1^. Blank samples were processed as for acetyltransferase. In the activity assay of Atpk2p and Afox2p, NADH was used as the cofactor. All other conditions were the same.

One unit of reductase activity was defined as the amount of 1 nmol NADPH oxidized to NADP^+^ in 1 min by 1 mg of dry permeabilized cells.

### Glucose assay

To determine glucose usage by the cells, a modified DNS assay (based on dinitrosalicylic acid—Miller 1959), which can indicate reducing sugars in culture medium, was performed. 100 μL DNS reagent (1% 3,5-dinitrosalicylic acid, 0.4 M NaOH, 0.4 M KOH, 30% Rochelle salt) was added to 100 μL of filtered and diluted culture supernatant, incubated in 95 °C for 10 min, cooled to room temperature and the solution was analyzed at 530 nm with a reference wavelength of 600 nm. The concentration of glucose was calculated from the measurement of standard dilutions.

### GC/MS analysis

GC/MS analysis was used to determine the concentration of 3-HB in the supernatant. 0.1–1 mL culture supernatant was filtered through a 0.4 μm syringe filter and lyophilized for 24 h. The dry supernatants were subjected to propanolysis as described by Riis and Mai (1988) but with modification. 1.5 mL 1,2-DCE and 1.5 mL *n*-propanol-HCl solution (4:1 v/v) was added to lyophilizate and incubated at 90 °C for 4 h. After cooling to room temperature the reaction mixture was extracted twice with triple distilled H_2_O and the lower organic phase was taken for analysis in a Clarus^®^ 680 GC combined with Clarus^®^ SQ 8 S MS (PerkinElmer, USA) equipped with Elite-624 column (PerkinElmer, 30 m × 0.5 mm, 1.4 μm). The GC program was set up as follows: initial 60 °C for 5 min, ramping at 10 °C min^−1^ to 200 °C and held for 2 min and then ramping at 5 °C min^−1^ to 235 °C and held for 10 min.

Commercial (R)-3-HB (Sigma-Aldrich) was used to construct a standard curve. The analysis was conducted in triplicate and results were analyzed by TurboMass 6.1 software.

### Enantiopurity analysis

To check enantiopurity of 3-HB, a d-3-hydroxybutyric acid assay kit (Megazyme, Ireland), which is specific for (R)-3-HB, was used. Samples were prepared as recommended by the manufacturer and the results were compared with GC/MS measurements. Each sample was analyzed 5 times and the results meaned to give the final values.

### Statistics

Two cultures were grown as independent experiments and measurements of enzymatic activity and GC/MS analysis were performed in triplicate. The final results are average values of the obtained data.

## Results

### (R)-3-HB shaking flask screening

Production of (R)-3-HB in *A. adeninivorans* was enabled by the overexpression of *β*-*ketothiolase* gene from different origins and *acetoacetyl*-*CoA reductase* gene from *C. necator* H16. Two endogenous *reductase* genes were functioning along with the *thiolase* gene from *C. acetobutylicum* (Table 2). The expression plasmids contained a YRC sequence (yeast rDNA integrative expression cassette) for homologous recombination or a YIC sequence (yeast integrative expression cassettes) for non-homologous recombination. The plasmids, Xplor2.4-atoB/thl/phaA/AKAT1/AKAT2/AKAT4/AKAT4_N10_/AKAT4_N17_-phaB and Xplor2.4-thl-ATPK2/AFOX2 were constructed with each containing both production genes flanked with strong constitutive *TEF1* promoter and *PHO5* terminator. The endogenous auxotrophic *AADE2* marker, which restores adenine synthesis pathway in *A. adeninivorans*, was used to select transformants. Plasmids were linearized with *Asc*I (YRC) or *Sbf*I (YIC) and transformed into the auxotrophic mutant, *A. adeninivorans* G1216, which is unable to grow on media that does not contain adenine (Fig. 1).

**Table 2:** Overexpressed genes for (R)-3-HB production

Gene	EMBL accession number	Organism
β-Ketothiolase		
*phaA*	LT608131	*C. necator* H16
*atoB*	LT608129	*E. coli* K12
*thl*	LT608130	*C. acetobutylicum* ATCC 824
*AKAT1*	LT608124	*A. adeninivorans* LS3
*AKAT2*	LT608125	
*AKAT4*	LT608126	
*AKAT4*_*N10*_	LT608127	
*AKAT4*_*N17*_	LT608128	
Acetoacetyl-CoA reductase		
*phaB*	LT608132	*C. necator* H16
*AFOX2*	LT632550	*A. adeninivorans* LS3
*ATPK2*	LT632551	

N10/N17 subscript represent protein with sequence started with depicted amino acid from N-terminal compare to wild type. All bacterial genes are codon optimized sequences. The constructed strains used in this article are the result of overexpression of one of the *thiolase* genes and one of the reductase genes

As well as the native endogenous thiolases, two versions of *A. adeninivorans* Akat4p, without a potential signaling peptide, were trialed. These variants were prepared by using another forward primer with an additional ATG start codon, processed and tested in parallel with the other thiolases.

The most productive strains in shake flask conditions were identified by incubating the transformants in YMM-glc-NO_3_ medium for 48 h and analyzing filtered supernatants by GC/MS. Except for G1216/YIC104-AKAT4-phaB, G1216/YRC104-thl-ATPK2 and G1216/YRC104-thl-AFOX2, all transgenic strains secreted (R)-3-HB into the culture medium (Table 3). The highest level of product was found in the G1216/YIC104-phaA-phaB clone 1 (0.56 g L^−1^). In contrast (R)-3-HB was not detected in negative control strain G1216/YIC104 (transformed with empty plasmid).

**Table 3:** (R)-3-HB production in shake flask using different media

Overexpressed genes			(R)-3-HB [g L^−1^]	
Thiolase	Reductase	Shaking flask (after 48 h)	Shaking flask (maximal level)	
			YMM	YPD
*atoB*	*phaB*	0.29 ± 0.03	0.88 [140 h]	2.74 [214 h]
*thl*		0.48 ± 0.01	2.15 [152 h]	4.87 [214 h]
*phaA*		0.56 ± 0.05	1.91 [140 h]	3.74 [214 h]
*AKAT1*		0.43 ± 0.01	0.34 [120 h]	2.76 [192 h]
*AKAT2*		0.38 ± 0.03	0.40 [144 h]	1.96 [216 h]
*AKAT4*		0.00	0.00	0.00
*AKAT4*_*N10*_		0.46 ± 0.05	0.81 [144 h]	3.22 [216 h]
*AKAT4*_*N17*_		0.51 ± 0.05	0.53 [120 h]	2.38 [192 h]
*thl*	*AFOX2*	0.00	0.00	0.00
	*ATPK2*	0.00	0.00	0.00
Negative control		0.00	0.00	0.00

The final row is *A. adeninivorans* G1216/YIC104 negative control strain transformed an empty plasmid

### Relationship between (R)-3-HB production and enzymes activities

Production of (R)-3-HB depends on the activity of β-ketothiolase and acetoacetyl-CoA reductase as well as other factors, e.g. enzymes involved in removing the CoA moiety from (R)-3-HB-CoA and secretion into the culture medium. To check the relationship between acid production and enzymes activities several tests were done. The best transformants of each of the gene combinations were chosen. Permeabilization rather than disruption of cells was used to retain the optimal environment for the enzymes.

**Fig. 2:**
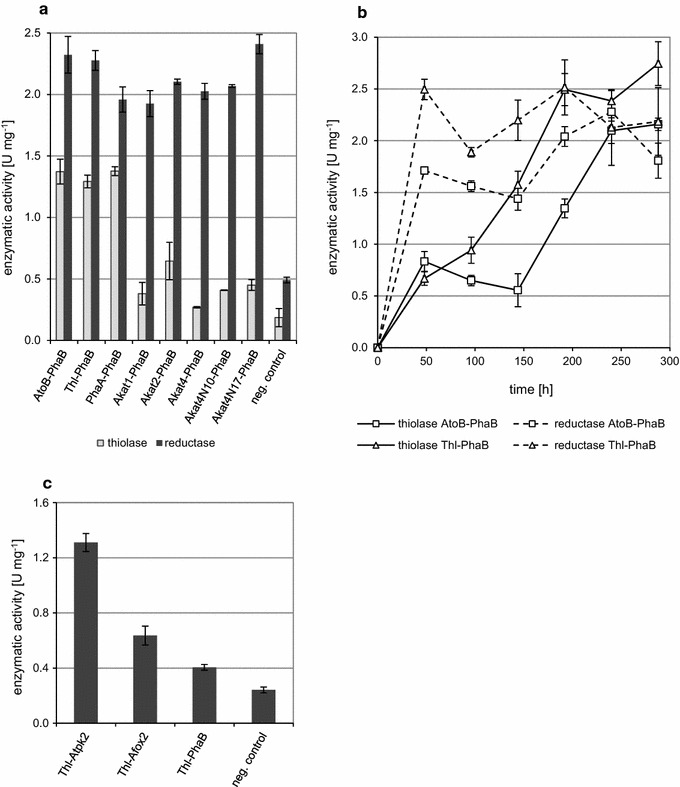
Enzyme activities of β-ketothiolases and acetoacetyl-CoA reductase; **a** 24 h cultivation on YMM-glc-NO_3_ (2% glucose) using all different strains, measured after 24 h; names under horizontal axis represent adequate genes overexpressed in *A. adeninivorans* cells; negative control—yeast transformed with empty expression plasmid. **b**—Enzymatic activities depends on cultivation time; G1216/YIC104-AtoB-PhaB (half-producing strain—triangle marker) and G1216/YIC104-Thl-PhaB (the best producing strain—square marker). **c** Enzymatic activities of different acetoacetyl-CoA reductases, usage of NADH as a cofactor instead of NADPH

**Fig. 3:**
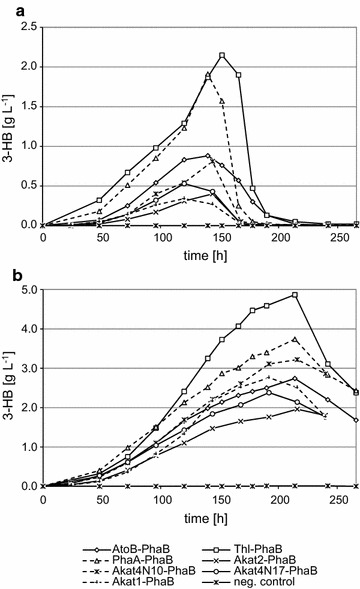
Time-course shaking flask culture for (R)-3-HB production; **a** YMM-glc-NO_3_; **b** YPD with additional 3% of glucose; negative control—yeast transformed with empty expression plasmid; names in legends represent adequate genes overexpressed in *A. adeninivorans* cells and is referred to both charts

The activity of thiolase was assayed for thiolysis activity. The results showed a difference between bacterial and yeast enzymes (Fig. 2a) with enzyme activities of recombinant bacterial thiolases being similar (1.38 ± 0.40, 1.37 ± 0.10 and 1.29 ± 0.05 U mg^−1^ for phaAp, atoBp and thlp, respectively), and while production of (R)-3-HB for G1216/YIC104-thl-phaB and G1216/YIC104-phaA-phaB is comparable, the amount of (R)-3-HB secreted by G1216/YIC104-atob-phaB is significantly lower. This phenomenon suggests that enzyme activity is not the only factor responsible for (R)-3-HB production. Activities of *A. adeninivorans* thiolases are 2-4-times lower than those reported for the bacterial enzymes expressed in this yeast with values between 0.27 ± 0.01 and 0.65 ± 0.15 U mg^−1^ for Akat4p and Akat2p respectively, which is associated with lower (R)-3-HB production in time course experiment (Fig. 3). The shorter versions of Akat4p without the predicted mitochondrial leader sequence (Akat4p_N10_, Akat4p_N17_) exhibited a higher enzymatic activity than native protein. Additionally G1216/YIC104-AKAT4-phaB (with a native mitochondrial thiolase) does not produce (R)-3-HB. The negative control has a relatively low activity of 0.19 ± 0.07 U mg^−1^ which corresponds to 13.5% of the maximal activity detected in the transformants.

The activity of acetoacetyl-CoA reductase, measured by the oxidation of NADPH, is similar for all transformants except for the negative control (transformed with an empty plasmid). The highest activity was exhibited by G1216/YIC104-AKAT4_N17_-phaB/1 with 2.41 ± 0.08 U mg^−1^ while the lowest activity of 1.93 ± 0.11 U mg^−1^ was seen in G1216/YIC104-AKAT1-phaB/2. The negative control had 0.49 ± 0.02 U mg^−1^ which corresponds to 20.4% of the highest detected activity. Enzyme activity was also seen in endogenous reductases Atpk2p and Afox2 using NADH as a cofactor instead of NADPH (Fig. 2c). The activity for G1216/YRC104-thl-ATPK2 was higher with 1.31 ± 0.07 U mg^−1^ compared to 0.64 ± 0.07 U mg^−1^ for G1216/YRC104-thl-AFOX2. Despite the enzymatic activity, both strains did not produce (R)-3-HB or (S)-3-HB. The negative control had 0.25 ± 0.02 U mg^−1^ which corresponds to 18.4% of the highest detected activity. As an additional negative control NADPH-dependent phaB reductase was tested. The strain G1216/YIC104-thl-phaB also exhibited a little of activity with 0.40 ± 0.02 U mg^−1^.

### Time course experiment

Changes of (R)-3-HB concentration in culture media were checked in shake flask time course experiments. The best strains of each gene combination were cultivated in YMM-glc-NO_3_ and YPD + 30 g L^−1^ glucose (to compensate sugar content). Each day samples were taken and (R)-3-HB and glucose concentration were measured; growth rate was determined by lyophilization of the cell pellet and determining the dry cell weight (dcw). In both media, the highest level of (R)-3-HB was observed with G1216/YIC104-thl-phaB clone 4 (Fig. 3). There was no production of (R)-3-HB in G1216/YIC104-AKAT4-phaB, G1216/YRC104-thl-ATPK2 and G1216/YRC104-thl-AFOX2 strains. In a rich medium, the concentration of the product rose to 4.87 g L^−1^ at 214 h with a productivity of 22.8 mg L^−1^ h^−1^. The decrease of (R)-3-HB level after this point is possibly caused by consumption of (R)-3-HB by *A. adeninivorans* as a carbon source. The results with the minimal medium were proportionally the same but the lower nutrient level resulted in a shorter incubation period and a lower productivity (maximum (R)-3-HB level at 152 h was 2.15 g L^−1^ and the productivity was 14.1 mg L^−1^ h^−1^). The secretion of (R)-3-HB started rapidly at the end of the exponential growth phase which means the production is biomass dependent rather than growth dependent i.e. production in idiophase when the carbon source has been depleted. This suggests that, as in other yeast, glucose is not directly converted to cytosolic acetyl-CoA, which is a necessary precursor for (R)-3-HB production (De Jong-Gubbels et al. 1995; Chen et al. 2012).

The enzymatic activities during extended cultivations were measured for the strains with the middle to highest (R)-3-HB production rates (Fig. 2b). The reductase in G1216/YIC104-thl-phaB reached 2.50 ± 0.10 U mg^−1^ after 48 h with constant activity until the end of cultivation. A similar situation was observed for G1216/YIC104-atob-phaB but with a lower maximal activity of 2.28 ± 0.09 U mg^−1^ after 240 h. Measurement of thiolase activity for G1216/YIC104-thl-phaB shows an incremental increase in its activity to 2.75 ± 0.21 U mg^−1^ at the end of incubation, with an increased rate between 48 h and 192 h. G1216/YIC104-atoB-phaB showed a steep rise in enzymatic activity starting at 144 h and reaching 2.16 ± 0.06 U mg^−1^ at the end of the cultivation at 288 h. These results can only partially explain the difference between (R)-3-HB productions by both strains. The threefold higher enzymatic activity of thiolase in contrast to only 1.75-fold higher (R)-3-HB production by G1216/YIC104-thl-phaB and G1216/YIC104-atoB-phaB shows that it is not only enzymatic activity that influences (R)-3-HB synthesis and secretion.

### (R)-3-HB utilization

One of the problems with (R)-3-HB synthesis by *A. adeninivorans* is degradation of this acid at the end of the synthesis stage. To check the influence of overexpressed genes on this phenomenon, an additional strain with an overexpressed *phaB* gene was constructed. G1216/YIC104-phaB, G1216/YIC104-thl-phaB and G1216/YIC104 (negative control) were used in time course experiment. After the medium shift, cultures were fed with different concentration of (R)-3-HB and cultivated for 48 h.

**Fig. 4:**
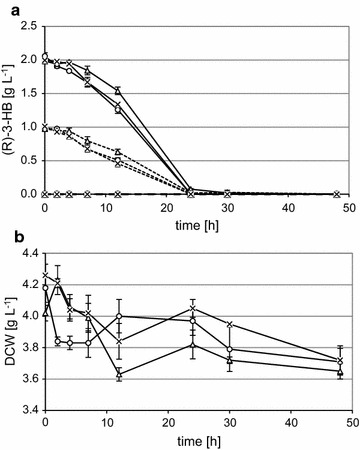
(R)-3-HB utilization. After the medium shift, cultures were fed with different (R)-3-HB concentrations. *Straight*, *dashed* and *dot-dashed lines* correspond to the initial 0.2, 0.1 and 0% of (R)-3-HB respectively. *Triangles*, *crosses* and *circles* correspond to strains G1216/YIC104-thl-phaB, G1216/YIC104-phaB and G1216/YIC104 (negative control) respectively. Cell masses were shown only for the 0.2% (R)-3-HB concentration because similar growth behavior was observed for the all strains. **a** (R)-3-HB concentration, **b** cell mass (dcw)

The results of (R)-3-HB concentration and cell mass (dcw) revealed that the overexpression of the *phaB* gene alone has no influence on (R)-3-HB utilization compared to the negative control (Fig. 4a). In G1216/YIC104-thl-phaB we observed a slightly lower rate of consumption but this is possibly due to ability of this strain to a parallel production of (R)-3-HB. Additionally, the decrease of cell mass during cultivation showed that carbon from (R)-3-HB is directed to secondary metabolism rather than to growth (Fig. 4b).

### Fermentation results

Time course analysis indicated that (R)-3-HB can be used by *A. adeninivorans* as a carbon source. To increase production levels and to prevent product consumption, nutrients were provided in fed-batch mode. Based on the results from the shake flask experiments, *A. adeninivorans* G1216/YIC104-thl-phaB and a modified yeast complex media were selected for the optimization of (R)-3-HB production.

**Fig. 5:**
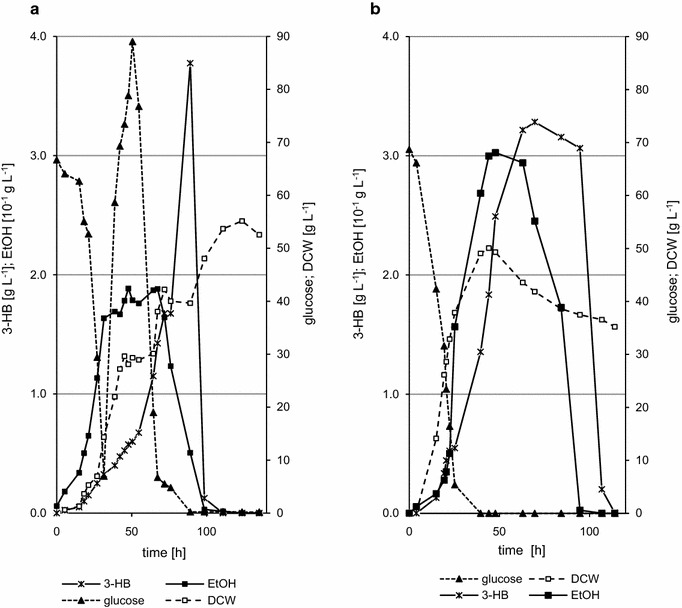
Production of (R)-3-HB by fed-batch cultivation of *A. adeninivorans* G1216/YIC104-Thl-PhaB: **a** under hypoxic condition (pO_2_ = 1%) with glucose feeding; **b** under aerobic condition (pO_2_ = 40%) with ethanol feeding. Feeding phase was started before initial glucose was completely consumed. During experiment the concentration of (R)-3-HB, glucose, ethanol and cell mass were measured

The first fermentations showed that in aerobic conditions (40% pO_2_), (R)-3-HB is consumed rapidly after glucose depletion, reaching maximum of 1.3 g L^−1^ of product after 26 h (data not shown) and production doesn’t occur again despite of glucose feeding. To try to counter the degradation of (R)-3-HB, hypoxic conditions (1% pO_2_) were used and the culture was fed with glucose and nitrogen, beginning before the depletion of the initial glucose. As expected, a longer growth lag phase occurred, 21 h compared to 5 h for growth in optimal conditions (data not shown). The end of the logarithmic phase occurred at 42 h with a specific growth rate of 0.066 h^−1^. Production of (R)-3-HB started from the beginning of log phase and rose steadily up to 76 h, which was when the initial glucose became depleted. From this point, the concentration increased from 1.66 g L^−1^ at 76 h to 3.76 g L^−1^ after 89 h of cultivation and attaining a productivity of 0.043 g L^−1^ h^−1^ (Fig. 5a). Longer cultivation however, caused (R)-3-HB degradation as was seen in shake flask experiments. The increase in (R)-3-HB production between 76 and 89 h was correlated with the use of ethanol by *Arxula* and once the ethanol was depleted, (R)-3-HB was then used as the carbon source.

To check if *Arxula* is able to produce (R)-3-HB from ethanol only, fed-batch fermentations were performed (Fig. 5b). The best conditions for ethanol feeding were establish by carrying out fermentation in aerobic conditions (pO_2_ = 40%). A higher oxygen level resulted in faster growth with the end of logarithmic phase occurring at 25 h. The production of (R)-3-HB started around 15 h at 0.13 g L^−1^ and increased to 3.28 g L^−1^ at 70 h with productivity of 0.047 g L^−1^ h^−1^. Despite the continuous ethanol feeding the level of (R)-3-HB remained constant. After feeding was complete, (R)-3-HB, as in other fermentations, was immediately degraded by *Arxula*. Additionally, despite the aerobic conditions, the amount of cell mass at the end of cultivation was lower than for hypoxic conditions and glucose feeding (35.2 g L^−1^ compare to 53.1 g L^−1^).

### Enantiopurity

Because of the specificity of acetoacetyl-CoA reductase, the final product was predicted to be the (R)-enantiomer (Tseng et al. 2009). GC/MS measurement using the standard column is not able to distinguish between (R) and (S) forms and thus to check enantiopurity of (R)-3-HB the results from GC/MS measurement and those from D-3-hydroxybutyric acid assay kit were compared. The sample was taken after 120 h cultivation of G1216/YIC104-thl-phaB in rich medium and concentration of (R)-3-HB was measured by both methods. The concentration of (R)-3-HB quantified by GC/MS and the kit assay was same (3.029 ± 0.044 and 3.027 ± 0.090 g L^−1^ respectivly). Because the kit assay can recognize only (R)-3-HB and there is no difference between both results, it confirms that (R)-3-HB produced by *A. adeninivorans* is a pure enantiomer. Additionally the (S)-3-HB negative control showed no response using the same assay kit.

## Discussion

Overexpression of *β*-*ketothiolase* and *acetoacetyl*-*CoA reductase* genes makes the yeast *A. adeninivorans* able to produce 3-hydroxybutyric acid. Additionally, the lack or low level of expression of endogenous enzymes able to produce (R)-3-HB and the overexpression of an enantiospecific reductase led to the secretion of enantiomerically pure (R)-3-HB.

Initial investigations, which included overexpression of different versions of *thiolase* genes from bacteria and *A. adeninivorans* and one *reductase* gene from *C. necator* H16 resulted in the production of (R)-3-HB by strains with all gene combinations, except G1216/YIC104-AKAT4-phaB which had a mitochondrial thiolase. The highest concentration was achieved with G1216/YIC104-thl-phaB. Additionally, strains with overexpressed *thl* gene and two endogenous *reductase* genes did not secrete 3-HB. Analysis of two different media for (R)-3-HB production in time course shake flask experiments revealed greater synthesis by all strains grown in a yeast complex medium (YPD) compared to growth in a minimal medium with nitrate (HMM-glc-NO_3_). Additionally, the degradation of (R)-3-HB occurred faster in the HMM-glc-NO_3_ medium. Those differences could be caused by more secondary metabolites for metabolism after the depletion of the initial glucose in YPD medium.

Comparison of expressed thiolases from different origins shows that the enzyme activity of the bacterial enzymes is at least twice as high than for *A. adeninivorans* enzymes and that enzymatic activity is not directly related to (R)-3-HB production. While G1216/YIC104-thl-phaB with bacterial thiolase exhibits the highest production and relatively high enzymatic activity, the G1216/YIC104-atoB-phaB strain that has a different bacterial thiolase, shows the highest activity, however it has a (R)-3-HB production level lower than that for G1216/YIC104-AKAT4_N10_-phaB containing an overexpressed *A. adeninivorans* gene. Those disparities have to be explained by other factors, for example, specificity of thiolases, its kinetic constants, optimal pH and temperature. Comparison of reductase enzymatic activity shows that bacterial phaBp has an almost twice the activity than endogenous Atpk2p and four times the activity of Afox2p. While strains with overexpressed *thiolase* and *phaB* genes are able to produce (R)-3-HB, the strains with *ATPK2* or *AFOX2* genes did not secrete either the (R) form or (S) form of 3-HB. This effect can be caused by compartmentalization of protein to different organelles. Bacterial phaBp is placed in cytosol, while Atpk2p and Afox2p are expressed in the mitochondria and peroxisome respectively. It is likely that inefficient transport of intermediates between cell organelles could impede (R)-3-HB production.

Two of the *A. adeninivorans* LS3 thiolases without potential mitochondrial signaling sequence were amplified and tested. Comparison of Akat4p_N10_ and Akat4p_N17_p with wild type Akat4p reveals interesting results. Enzymatic activities of shorter versions are slightly higher than for the wild type. Production of (R)-3-HB is relatively high for G1216/YIC104-AKAT4_N10_-phaB and G1216/YIC104-AKAT4_N17_-phaB, especially for the former, while the strain G1216/YIC104-AKAT4-phaB does not exhibit any secretion of the product. These results show that cytosolic targeting of protein is necessary for (R)-3-HB production. Additionally, enzymes with deleted signaling sequence retained their enzymatic activity, and were able to synthesis of (R)-3-HB. This strategy could be employed in other applications such as express mitochondrial or peroxysomal enzymes in cytoplasm. Further analysis of shake flask cultures showed an increase in secretion with longer cultivation periods (up to 214 h). Increased late production can be hypothetically explained by two factors. Firstly, a flux of acetyl-CoA at the beginning of cultivation on glucose is used in the mitochondria for energy production (Krivoruchko et al. 2015) and due to oleaginous nature of *Arxula*, used for fatty acid synthesis (Ratledge 2002). After glucose depletion fatty acids can be reduced and more acetyl-CoA shifted to (R)-3-HB synthesis pathway. Additionally the correlation between ethanol and (R)-3-HB levels revealed that acetyl-CoA coming from ethanol utilization is responsible for high secretion peak and it seems to be the main source of (R)-3-HB precursor. This effect can be explained by fact that acetyl-CoA from ethanol is localized in cytoplasm (De Jong-Gubbels et al. 1995) and can be directly converted by β-ketothiolase. Secondly, the enzymes responsible for secretion of (R)-3-HB in *A. adeninivorans* LS3 are currently unknown and therefore cannot be influenced. Late (R)-3-HB production could also indicate that this excretion system is inefficient. Additionally, it is unknown whether secretion mechanism is active or non-active. There are more than 10 genes in *A. adeninivorans* LS3 which could express proteins involved in this system, but most of them are located in mitochondria or peroxisomes. Nevertheless, there exist at least three transferases which are localized in cytoplasm. Two of them are constitutively expressed and one is induced by oleic acid. Additionally, these enzymes exhibit high activity for the transfer of the CoA moiety to succinyl-CoA (data not published). Liu et al. (2007) obtained up to 12 g L^−1^ of (R)-3-HB in *E. coli* using another system with thioesterase II (tesB) for removing CoA from (R)-3-HB-CoA, thus facilitating secretion of the acid into the medium. A similar protein occurs in *A. adeninivorans* LS3 and is analogous to *Pte1* from *S. cerevisiae* (Maeda et al. 2006) but both are targeted to peroxisomes. To improve production of (R)-3-HB in *A. adeninivorans* by genetic modification, an endogenous secretion system could be overexpressed or an exogenous secretion system could be expressed.

Utilization of (R)-3-HB as a carbon source cannot be explained by as an effect of overexpressed genes. The negative control strain and strain with overexpressed *acetoacetyl*-*CoA reductase* gene have a similar (R)-3-HB utilization behavior and (R)-3-HB producing strain G1216/YIC-104-thl-phaB catabolizes acid slightly slower but this effect can be explained by parallel production of (R)-3-HB. These results confirmed that (R)-3-HB can be used by *A. adeninivorans* as a carbon source and that this process currently cannot be controlled.

To boost the production of (R)-3-HB the controllable conditions of fed-batch cultivation was employed. Optimization of fermentation conditions using different aeration rates revealed that (R)-3-HB secretion is highly dependent on oxygen level. Fully aerobic conditions lead to rather fast (R)-3-HB production but also rapid product consumption. Three-fold higher synthesis level occurs for hypoxic conditions (pO_2_ = 1%) but a longer incubation period was required (89 h compare to 26 h). However under these conditions degradation of acid also occurred. In both cases after depletion of basic carbon sources, endogenous ethanol is consumed and causes an increase in production, however after ethanol is completely catabolized, (R)-3-HB is rapidly consumed by *Arxula* and it becomes difficult to maintain a high production level. Using ethanol as the main carbon source did not improve the secretion level, which could mean there is a maximal level of (R)-3-HB in fed-batch fermentation. Despite using controlled hypoxic conditions and higher glucose or ethanol concentrations, the level of (R)-3-HB never exceeded the concentration obtained in shake flask cultures. Here we have demonstrated that the non-conventional yeast *A. adeninivorans* is able to produce enantiomerically pure (R)-3-HB up to 4.87 g L^−1^ in shaking flasks and 3.76 g L^−1^ in fed-batch culture. In future work, we will attempt to increase the secretion level and decrease the cultivation period. Genetic engineering an alternative membrane transport system and the use of different carbon sources appear to be promising strategies for this enhancement.

## Abbreviations

(R)-3-HB: (R)-3-hydroxybutyric acid
PHB: polyhydroxybutyrate
PHB-V: poly(hydroxybutyrate-co-hydroxyvalerate)
CoA: coenzyme A
GC/MS: gas chromatography/mass spectrometry
DCE: 1,2-dichloroethane
YRC: yeast rDNA integrative expression cassette
YIC: yeast integrative expression cassette
dcw: dry cell weight
